# What Does Sarcopenia Have to Do with Nonalcoholic Fatty Liver Disease?

**DOI:** 10.3390/life14010037

**Published:** 2023-12-25

**Authors:** Katarzyna Ferenc, Sara Jarmakiewicz-Czaja, Rafał Filip

**Affiliations:** 1 Institute of Medicine, Medical College of Rzeszow University, 35-959 Rzeszow, Poland; kferenc@ur.edu.pl; 2 Institute of Health Sciences, Medical College of Rzeszow University, 35-959 Rzeszow, Poland; 3 Department of Gastroenterology with IBD Unit, Clinical Hospital No. 2, 35-301 Rzeszow, Poland

**Keywords:** fatty liver, inflammation, insulin resistance, sarcopenia

## Abstract

Nonalcoholic fatty liver disease (NAFLD) is one of the most common causes of chronic liver disease. As the second stage of developing steatosis, nonalcoholic hepatitis (NASH) carries the risk of fibrosis, cirrhosis, and hepatocellular carcinoma. Sarcopenia is defined as a condition characterized by a decrease in muscle mass and functional decline. Both NAFLD and sarcopenia are global problems. The pathophysiological mechanisms that link the two entities of the disease are insulin resistance, inflammation, nutritional deficiencies, impairment of myostatin and adiponectin, or physical inactivity. Furthermore, disorders of the gut-liver axis appear to induce the process of developing NAFLD and sarcopenia. The correlations between NAFLD and sarcopenia appear to be bidirectional, so the main objective of the review was to determine the cause-and-effect relationship between the two diseases.

## 1. Introduction

Nonalcoholic fatty liver disease (NAFLD) is one of the most prevalent liver diseases worldwide [1]. It is estimated that a quarter of the population is affected [2]. NAFLD is determined by liver steatosis that is not related to alcohol consumption [3]. Due to the severity of the disease, two stages of the disease can be distinguished [4]. The first is nonalcoholic fatty liver (NAFL), which is not associated with inflammation and fibrosis [5]. The next stage is nonalcoholic steatohepatitis (NASH). It is associated with inflammatory infiltration, the possibility of developing fibrosis and cirrhosis, and hepatocellular carcinoma (HCC) [6]. The term MAFLD, which describes steatohepatic disease associated with metabolic dysfunction, is increasingly appearing in scientific reports. Metabolic dysfunction-associated fatty liver disease (MAFLD) is said to emphasize the association between the coexistence of liver steatosis and various components of metabolic syndrome. Importantly, MAFLD, unlike NAFLD, does not exclude alcohol abuse [7].

Sarcopenia is a condition characterized by a decrease in muscle mass, a decrease in muscle function, and a loss of physical fitness. Sarcopenia can be divided into primary and secondary. Primary sarcopenia is closely related to the physiological process of aging. Secondary sarcopenia, on the other hand, is associated with chronic diseases but also with dietary errors and physical inactivity [8,9]. It is estimated to occur in 11–50% of patients older than 80 years of age. The global trend toward aging has made sarcopenia a worldwide problem [10]. A study was conducted on 15,132 individuals from the Korean population to determine the association between NAFLD and sarcopenia. Sarcopenia has been shown to be associated with a higher risk of NAFLD, regardless of the presence of obesity [11]. Another study was conducted in the United States of America (USA) with 11,325 participants. The researchers identified NAFLD based on the presence of hepatic steatosis on ultrasound. The prevalence of NAFLD was shown to be more common in patients with sarcopenia than without sarcopenia [12]. In addition, it appears that an increase in muscle mass may have an effect on inhibiting the development of NAFLD [13]. When analyzing all the data, it is difficult to determine whether sarcopenia is a result of NAFLD or its risk factor. Therefore, the main purpose of the review is to identify factors that are associated with both sarcopenia and non-alcoholic fatty liver disease.

## 2. Nonalcoholic Fatty Liver Disease (NAFLD)

NAFLD is one of the most frequently diagnosed chronic liver diseases [14,15]. It is considered the leading cause of mortality, taking into account all liver-related pathologies [16]. NAFLD is a broad and collective term that describes a spectrum of liver diseases resulting from causes other than excessive alcohol consumption [17]. NAFLD includes liver steatosis in >5% of all hepatocyte cells. Liver steatosis itself is referred to in medical nomenclature as nonalcoholic fatty liver (NAFL). The next stage in which inflammation occurs along with liver cell damage is called nonalcoholic steatohepatitis (NASH) [18]. NASH can eventually lead to the development of organ fibrosis and cirrhosis [19].

Importantly, the liver is an organ that accounts for 15% of oxygen consumption in the human body. It follows that hepatocytes are cells rich in mitochondria [20]. It has been observed that in mouse models with NAFLD, there is a decrease in mitochondrial ATP-synthesizing respiration. This appears to occur because the mitochondria are unable to oxidize sufficient fatty acids [21,22]. Impaired mitochondrial function can induce oxidative stress. Consequently, it promotes inflammation and predisposes to NAFLD [23]. It appears that preventing NAFLD progression and restoring liver function can be achieved by focusing on improving mitochondrial activity. There are recent indications that the ketogenic diet can induce improvements in mitochondrial function through stimulation of mitochondriogenesis and bioenergetic pathways [24,25].

In the final stage, impaired liver function can induce liver failure, which requires organ transplantation [26]. In 2009, NASH represented about 10% of liver transplants in the United States [27]. Approximately 30–40% of patients with NAFLD have been reported to develop NASH [28]. Furthermore, patients with NAFLD have an increased risk of extrahepatic complications, including cardiovascular disease and malignancies [29,30]. Currently, the prevalence of NAFLD is estimated to be around 25% in the general population [31]. The number of patients with NAFLD in the United States is estimated to increase from 83.1 million in 2015 to 100.9 million in 2030 [32]. The analyses show that NAFLD can develop in 70% of overweight individuals and up to 90% of those with established obesity [33]. Asians, on the other hand, have been found to accumulate fat in hepatocytes more rapidly than normal-weight individuals [34]. The number of patients with NASH is also expected to increase due to the aging process, as is the expected increase in the prevalence of diabetes among the elderly [32]. Furthermore, the percentage of children diagnosed with hepatic steatosis has increased in recent years [35].

Recently, researchers have pointed out that the name NAFLD does not emphasize the role of the metabolic syndrome in the overall pathogenesis as well as the complications of liver disease. Therefore, a new definition has been proposed, which is MAFLD, or metabolic steatohepatic disease. This definition defines an independent disease entity and excludes the criterion of alcohol abuse [36]. Many studies show a reciprocal correlation between NAFLD and metabolic disorders, which indicates that NAFLD may be a cause but also a consequence of extrahepatic metabolic diseases [37,38]. In patients with established NAFLD, it is very common to find the coexistence of one or more components of metabolic syndrome (MS), such as dyslipidaemia, hypertension, insulin resistance (IR), and diabetes [39,40]. It is widely believed that NAFLD is one of the hepatic manifestations of MS [41]. Importantly, the mechanism of interaction between MS and NAFL may be bilateral. Consequently, NAFLD increases the possibility of MS [42]. The coexistence of NAFLD with MS is correlated with an increased risk of cardiovascular disease and type 2 diabetes. On the contrary, no such correlations were found in patients with NAFLD without concomitant MS [43]. On the other hand, Baratta et al. showed that patients with liver steatosis have an increased risk of cardiovascular disease, and this risk is further increased in patients with already established organ fibrosis [44]. Importantly, patients without established excess body weight have also been shown to have a higher rate of cardiovascular events [45]. Another study showed that NAFLD, regardless of predisposing factors, increases the risk of myocardial infarction [46]. In addition, there are meta-analyses that confirm that the presence of NAFLD is associated with cardiovascular disease [47,48].

There is a lack of approved drugs to treat NAFL and NASH [49]. The European Association for the Study of the Liver (EASL) recommends that patients make lifestyle modifications. Focusing especially on physical activity and changes in eating habits. In recent years, the Mediterranean diet has been proposed and may be beneficial for patients with NAFLD [50]. Adherence to a Mediterranean diet for 6 months resulted in a decrease in intrahepatic fat and an improvement in the state of the MS trait [51]. The reduction in weight in overweight or obese individuals with established NAFLD resulted in a greater improvement in liver function and increased insulin sensitivity compared to healthy overweight individuals [52]. Interventions such as lifestyle changes appear to be the most important and effective for preventing and controlling NAFL without developing NASH and fibrosis [53]. A weight loss of 7–10% appears to have beneficial effects in patients with NAFLD, regardless of diet composition [54]. However, many scientific societies recommend the Mediterranean diet as the recommended dietary pattern [55]. Softic et al. show that fructose has a greater effect on obesity and insulin resistance compared to glucose. Especially when combined with a high-fat diet [56]. In addition, it appears that a high-carbohydrate or fat-only-focused diet is not sufficient to induce NAFLD. It has been suggested that it is the combination of different nutrients that stimulates the induction of liver disease [57]. In addition, the introduction of physical activity helps maintain muscle mass and strength, especially in middle-aged and older individuals [58].

Therefore, it is necessary to undertake research that will help determine the exact quantitative and qualitative composition of diet and physical activity intensity that will enable a reduction in the risk of NAFLD while also halting the progression of the disease.

## 3. Sarcopenia

Sarcopenia is defined as muscle failure and is defined by a low index of measurements such as strength, quantity, quality of muscle, and physical capacity [59]. Sarcopenia is divided into primary and secondary. The primary is related to age and is a natural process such as aging, and no other significant cause can be identified [60]. Secondary, on the other hand, is due to systemic diseases, physical inactivity, or nutritional errors [61]. In addition, sarcopenic obesity is referred to in the literature. It is defined as the co-occurrence of obesity and sarcopenia [15]. Sarcopenia is a multifactorial disease. The main risk factors for sarcopenia include older age, female sex, low levels of physical activity, and the presence of chronic diseases [62]. Oxidative stress, chronic inflammation, inadequate caloric intake, and neuromuscular junction degeneration overlap to cause the progression of sarcopenia [63,64]. Decreased muscle mass plays an important role in the induction of IR and metabolic syndrome. Sarcopenia is often associated with cardiometabolic disorders such as cardiovascular disease and diabetes [65]. Han et al. showed that both sarcopenia and cardiovascular disease can be associated with similar risk factors such as hypertension, diabetes, dyslipidemia, and metabolic syndrome [66]. The effect of sarcopenia and the amount of muscle mass in liver disease is also increasingly being studied [67].

It is estimated that sarcopenia is diagnosed in about 29% of elderly people living in healthcare facilities, with those aged ≥80 years estimated to have sarcopenia in 11–50% of them [68]. Sarcopenia contributes to an increased risk of falls and disability, increased hospitalizations, and mortality [69,70]. In some sense, primary sarcopenia is inevitable, but its severity depends on the amount of physical activity, hormonal balance, the ability to synthesize and regenerate proteins, or early developmental influences [71]. Sarcopenia appears to be a physiological process, as it begins between the ages of 30 and 40 and is found to increase after the age of 60 [72]. The physiological changes that occur with aging can have important implications in terms of decreased muscle mass and reduced functionality. These changes include low-level chronic systemic inflammation and increased concentrations of reactive oxygen species (ROS) [73]. Ageing causes an increase in oxidative stress, which negatively affects mitochondrial function [74]. When mitochondrial function and structure are disrupted, redox balance is impaired. As a consequence, cell function is impaired, and, thus, health deteriorates [75]. Ageing is associated with ROS production but also with impaired endogenous antioxidant enzyme production in muscle and brain. This process can induce sarcopenia [76,77,78]. Additionally, elevated inflammatory mediators are found in the elderly. These include interleukin 6 (IL-6), tumor necrosis factor α (TNF-α), and C-reactive protein (CRP) [79]. In addition, naturally occurring aging causes an imbalance between the anabolic and catabolic mechanisms of muscle proteins [80]. Insufficient calorie and protein intake leads to a reduction in muscle mass and function [72]. In women after menopause, the concentration of sex hormones decreases, among other estrogen [81]. Patients with sarcopenia often experience a reduced quality of life, most often resulting from a decrease in physical fitness. This lower quality of life can, therefore, hinder patient communication [82]. Hsu et al. showed that in adults with established obesity, a high-protein diet combined with physical activity can improve strength, muscle function, and exercise capacity [83]. Similarly, Seo et al. showed that resistance training in older women with diagnosed sarcopenia improves muscle quality and functional capacity [84]. Resistance training appears to be used in prevention and as a form of therapy for sarcopenia in adults and the elderly [85,86,87].

## 4. NAFLD and Sarcopenia: Common Factors

Patients with sarcopenia have poorer recurrence-free survival (RFS) in early-stage intrahepatic recurrent hepatocellular carcinoma [88]. Its severity increases with the progression of the underlying disease. In patients with cirrhosis, its prevalence is estimated to be around 60% [89,90]. Its incidence depends on the ethical background, the severity of liver disease, and the selected criteria for its diagnosis [91]. Furthermore, sarcopenia has been identified as a predisposing factor to the severity of NAFLD [92]. Its presence is associated with an increased risk of NAFLD, but also with the appearance of advanced organ fibrosis [93,94]. Lee et al. came to similar conclusions. In their study, they showed that sarcopenia is correlated with organ fibrosis in patients with NAFLD. Importantly, this association was independent of IR and obesity [95]. Similarly, Koo et al. observed a correlation between fibrosis, NAFLD, and sarcopenia, further indicating that this relationship was not dependent on systemic inflammation [96]. Tantai et al. found that sarcopenia associated with cirrhosis could increase the risk of death by two times. Furthermore, mortality increased with the severity and duration of sarcopenia [97]. Studies show that the presence of sarcopenia in patients with cirrhosis generates an increased incidence of infections, prolonged hospitalization after organ transplantation, and increased hyperammonemia and visible liver encephalopathy [98,99,100]. In addition, sarcopenia generates metabolic disorders that include impaired glucose tolerance, impaired ammonia, and amino acid metabolism. Also, it affects bone structure. Sarcopenia and osteoporosis that occur at the same time are defined as osteosarcopenia [101]. Saeki et al. studied the prevalence of osteosarcopenia in 291 patients with chronic liver disease. Osteosarcopenia was found in 16.8% of patients [102]. Patients with coexisting sarcopenia and NAFLD have reduced creatinine production, which causes problems in estimating renal function in these patients [103]. In cirrhosis, factors such as hyperammonemia, hypogonadism, and branched-chain amino acid (BCAA) deficiency appear to influence the development and progression of sarcopenia [104]. In addition, nutritional disorders, insulin resistance, lipid disorders, and a disturbed gut microbiota [105]. Both the risk of NAFL and sarcopenia increases with age, and both conditions are a major public health problem and a burden on health care [98,106]. Many studies indicate associations between the presence of sarcopenia and NAFLD. However, they very often lack an analysis of factors that may interfere with the final results. Undoubtedly, lifestyle, which is defined by factors such as nutrition and physical activity but also economic and social factors, has a huge impact on the final analyses. The complexity of these factors makes them difficult to measure unambiguously. As a result, it is difficult to adjust to them. The research is presented in Table 1.

### 4.1. Insulin Resistance

**Figure 1:**
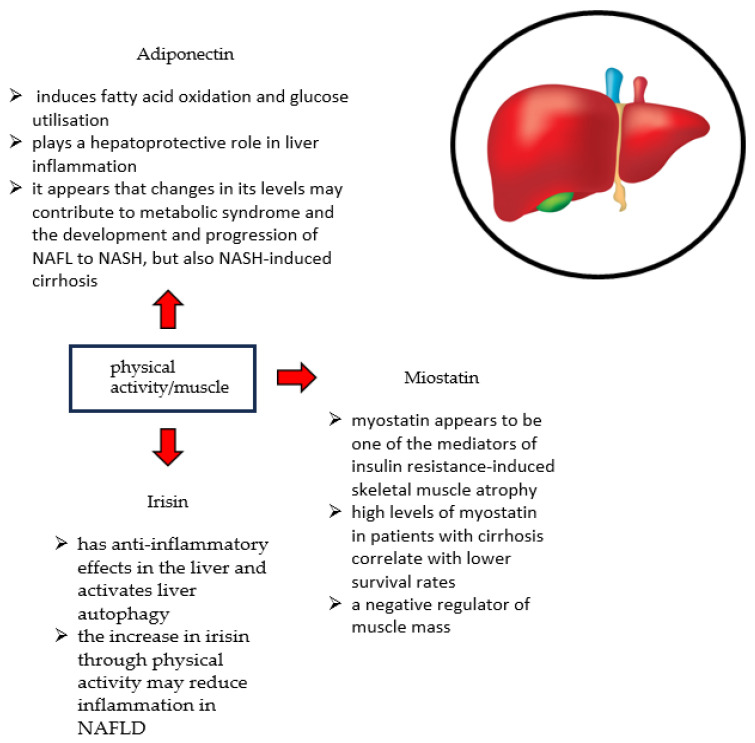
The relationship between muscle, myokines, and liver.

NAFLD and sarcopenia share common pathophysiological mechanisms. These include IR, systemic inflammation, impairment of myostatin and adiponectin function, catabolic factor production, nutritional deficiencies, and physical inactivity [12,110]. This is shown in Figure 1.

Studies show that IR is a pathological condition that can induce NAFLD and sarcopenia [111,112,113]. IR is a situation in which the physiological response of target skeletal muscle cells to insulin is disrupted and the body’s carbohydrate metabolism is impaired, thus enhancing the development of sarcopenia. IR appears to induce sarcopenia by affecting various physiological processes in the body. These include decreased synthesis of skeletal muscle proteins and increased catabolism, increased expression of forkhead box O (FOXO) family proteins, and the autophagy process that occurs in skeletal muscle [114]. Patients with NAFLD appear to be prone to sarcopenia despite a high body mass index (BMI) [15]. Furthermore, impaired gluconeogenesis caused by hyperinsulinemia promotes proteolysis and decreases protein synthesis. This situation, with age, can promote sarcopenia [115]. On the other hand, a decrease in muscle mass exacerbates IR. This is due to the fact that skeletal muscle is the primary insulin-responsive organ [116]. Hyperinsulinemia associated with IR causes inhibition of β-oxidation of fats in the liver and an increase in the binding protein of the sterol regulatory element 1c (SREBP-1c). This results in the accumulation of triacylglycerols and free fatty acids in the organ [117]. Smith et al., in their study, showed that de novo lipogenesis in the liver is responsible for the regulation of intrahepatic triglycerides and that increased glucose and insulin levels induce de novo lipogenesis [118]. Importantly, NAFLD can also induce IR. It appears that this may be due to the action of hepatokines such as fibroblast growth factor (FGF)-21 and fetuin-A, which are produced by the liver in response to oxidative stress [119].

### 4.2. Myosteatosis

Patients with liver disease, especially cirrhosis, can simultaneously experience a situation in which fat mass increases and skeletal muscle mass is reduced. This condition can be referred to as “sarcopenic obesity”. It appears that inflammation, insulin resistance, and vitamin D deficiency may be associated with sarcopenic obesity in the Korean population [120]. In addition, sarcopenic obesity is associated with higher morbidity and mortality than these individuals separately [15]. Importantly, loss of muscle mass characterized by both a decrease in muscle size and an increase in intermuscular fat is defined as myosteatosis. Infiltration of adipose tissue into skeletal muscle is associated with metabolic abnormalities and reduced muscle mobility and strength. Its severity increases with the age of the patient [121,122]. The presence of myosteatosis is correlated with a high mortality risk, but also with IR [123]. Hsieh et al. showed that advanced myosteatosis is associated with the early progression of NASH and liver fibrosis [124]. Nachit et al. propose myosteatosis as a non-invasive marker for the detection of NASH [125].

### 4.3. Inflammation

Sarcopenia and NAFLD are also related to systemic inflammation. The accumulation of lipids promotes the secretion of pro-inflammatory cytokines from adipose tissue. This results in ROS induction. This occurs through impaired mitochondrial function, which consequently initiates lipid peroxidation. Interleukin-6 (IL-6), tumor necrosis factor-α (TNF-α), and transforming growth factor-β (TGF-β) are among the cytokines that generate low-grade chronic inflammation [109,126]. Patients with NAFLD also show elevated levels of TNF-α. TNF-α, in turn, is responsible for stimulating nuclear factor κB, which is involved in the development of NAFLD but also induces muscle catabolism [127,128]. In addition, TNF-α participates in the activation of de novo lipogenesis, which ultimately causes lipid accumulation in the liver [129]. In sarcopenia, the main pro-inflammatory cytokines include TNF-α, IL-6, and interleukin-1 (IL-1) [130]. Hong et al. showed that patients with sarcopenia have higher levels of CRP compared to patients without sarcopenia. In addition, they found that CRP levels showed a negative correlation with the index of liver function and skeletal muscle mass. These findings may indicate that inflammation is involved in the pathogenesis of NAFLD as well as sarcopenia [107]. Patients with NAFLD have excessive oxidation of free fatty acids (FFA); consequently, this promotes the formation of ROS. These, in turn, cause lipid peroxidation and the production of pro-inflammatory cytokines. In addition, liver-produced hepatokines are produced by the liver (e.g., fetuin A and B, selenoprotein P), which, due to their multidirectional functions, affect IR, lipid metabolism, protein catabolism, and sarcopenia. These broad-spectrum functions of hepatokines may explain the relationship between adipose tissue, muscle tissue, and the liver [131].

### 4.4. Vitamin D

Vitamin D deficiencies may increase the risk of developing sarcopenia [132]. Vitamin D is responsible for the increase in muscle fiber size, but it also increases their strength and endurance [133]. Furthermore, it is involved in cell differentiation and the proper functioning of the immune system, as well as in the regulation of cardiovascular and calcium-phosphate homeostasis [134]. In the elderly, vitamin D deficiency is common and a worldwide problem. In this group of people, it is usually due to low exposure to the sun and chronic diseases such as renal failure and malabsorption [135]. Recently, a correlation has been confirmed between vitamin D deficiency and the progression of fibrosis in chronic liver disease [136,137,138]. The prevalence of vitamin D deficiency is estimated to be found in 64% to 92% of patients with chronic liver disease [139]. The causes of these deficiencies appear to include a lower supply of exogenous vitamin D (both from the diet and low sun exposure), impaired absorption, decreased synthesis of vitamin D binding protein (VDBP), but also impaired hydroxylation in the liver and increased catabolism of 25(OH)D [140]. Izadi et al. showed an inverse relationship between 25(OH)D levels and aspartate aminotransferase (AST) and alanine aminotransferase (ALT) [141]. In addition, Wang et al. showed a positive correlation between vitamin D levels and irisin levels in women with sarcopenia [142]. Studies on vitamin D supplementation in patients with NAFLD are conflicting, so further research is needed in this area [143,144,145,146].

### 4.5. Physical Activity

Physical activity induces the production of anti-inflammatory cytokines while inhibiting the production of pro-inflammatory cytokines. Physical exertion also increases muscle protein synthesis and glucose uptake, which reduces the risk of sarcopenia [147]. On the contrary, lack of exercise can induce chronic inflammation, IR, and oxidative stress, resulting in disease exacerbation in patients with NAFLD and sarcopenia [148]. Additionally, physical inactivity results in reduced energy consumption and reduced muscle mass. The result is a fatty liver and obesity [149]. Furthermore, physical activity can contribute to a decrease in the secretion of fetuin A, which is responsible for the promotion of IR in the muscle and liver, but also increase the secretion of the inhibitor myostatin [150]. Increasing physical activity in sarcopenic patients can help reduce the risk of progression of IR [147]. Iwanaga et al. showed that physical activity based on electrical neuromuscular stimulation reduces the severity of IR by lowering IL-6 and selenoprotein levels [151]. Physical activity associated with building muscle mass plays an important role in reducing the risk of chronic diseases. Physical training has the potential to act as a therapy for lifestyle-related diseases. These include cardiovascular disease, cancer, type 2 diabetes, and dementia [152]. In addition, exercise-induced IL-6 can promote and enhance lipolysis [153]. Given these mechanisms, analyses confirm that low physical activity is associated with a higher risk of NAFLD [118,154]. Ageing, fat, and IR accumulation alter the signaling pathways for growth hormone (GH) and insulin-like growth factor 1 (IGF-1). Consequently, this leads to a decrease in muscle mass synthesis [155]. Cabrera et al. showed that induced NAFLD mice show an association of reduced IGF-1 with reductions in muscle mass and muscle strength. The authors also suggest a link between reduced IGF-1 and the pathogenesis of NAFLD-associated sarcopenia [108]. The study by Foong et al. showed that the intensity and amount of physical activity had an independent dose-response relationship with lower limb strength and percent lean body mass. These results suggest that increased physical activity in the elderly is necessary to maintain body weight with age [156]. In addition, in another study, Foong et al. suggest that increased physical activity may be necessary for weight reduction in older adults [157]. Importantly, physical activity is a key factor in the development of sarcopenia. Physical activity is responsible for skeletal muscle synthesis; its absence can result in the development of IR [15,62]. Furthermore, physical activity can be a major confounding factor in many studies, so it seems important to carefully analyze its presence and intensity in the patients studied.

### 4.6. Myokines

Skeletal muscles not only belong to the musculoskeletal system but also play an important secretory role [158]. Skeletal muscles secrete myokines, including Il-6, irisin, myostatin, and adiponectin. They play a role in the regulation of glucose and fatty acid metabolism. Disturbed levels of these myokines resulting from decreased muscle mass can cause fat accumulation in the liver [93]. The relationship between muscle, myockines, and liver is shown in Figure 1.

In patients with liver disease, hepatocyte dysfunction, impaired urea cycle, constipation, and intestinal dysbiosis contribute to hyperammonemia. The increased absorption of ammonia by skeletal muscle contributes to the development of sarcopenia. Muscle hyperammonemia results in increased myostatin expression and decreased activation of nuclear factor kappa B (Nf-B). As a result, impaired muscle protein synthesis occurs [159]. Myostatin is a negative regulator of muscle mass, which plays a key role in the development and maintenance of skeletal muscle mass [160]. Mutations or genetic disorders resulting in myostatin atrophy cause skeletal muscle hypertrophy [161]. Myostatin appears to be one of the mediators of insulin resistance-induced skeletal muscle atrophy [103,162]. In addition, in patients with cirrhosis, significantly elevated levels of ammonia can increase myostatin expression. Hyperammonemia appears to be an important inducer of sarcopenia by affecting the increase in myostatin [163]. Nishikawa et al. showed that high myostatin levels in patients with cirrhosis were correlated with lower survival rates [164]. In contrast, irisin is a myokine that is produced in response to exercise. It is responsible for fat-browning and thermogenesis [165]. Additionally, it exhibits anti-inflammatory effects in the liver and activates liver autophagy [166]. In addition, Zhao et al. showed that irisin is associated with sarcopenia in patients with cirrhosis [167]. Kosmalski et al. suggest that irisin can be used as a diagnostic marker for NAFLD, as it is associated with biochemical and anthropometric parameters related to liver function [168]. Wu et al. showed that irisin treatment can reduce age-related skeletal muscle fibrosis [169]. On the contrary, Zhu et al. have shown that increasing irisin levels through physical activity can reduce inflammation in NAFLD [170]. Adiponectin is one of the most common adipokines in human plasma. It is produced and secreted primarily by white adipose tissue [171]. Adiponectin induces fatty acid oxidation and glucose utilization [172]. In addition, it exhibits anti-inflammatory effects. In liver inflammation, it plays a hepatoprotective role [173]. It appears that changes in their levels can contribute to the development of MS, the development and progression of NAFL to NASH, and also NASH-induced cirrhosis [174,175]. The myokines described above are shown in Table 2.

### 4.7. Intestinal Microbiota

The intestine is directly connected to the liver through the portal vein [176]. These organs interact with each other through multiple pathways and are thus involved in the induction and progression of many diseases [177]. Intestinal bacteria and their metabolites can enter the liver through the portal vein, through which they can influence liver disease processes [178]. Dysfunction of the gut-liver axis contributes to the pathogenesis of NAFLD through processes such as disruption of the intestinal barrier and intestinal translocation, as well as the inflammatory response of the liver [179]. Disruption of the gut microbiota in cirrhosis contributes to hyperammonemia. This, in turn, plays a key role in the induction of sarcopenia and IR [180]. Furthermore, in cirrhosis, intestinal dysbiosis is associated with reduced intestinal bacterial diversity, reduced antioxidant activity, and endotoxemia. This in turn is associated with chronic inflammation, mitochondrial dysfunction, and IR, which can cause the progression of cirrhosis and sarcopenia [181]. Furthermore, recent indications suggest that amino acids synthesized by the gut microbiota may be involved in the occurrence of sarcopenia [182]. Obese people are found to have an increased risk of dysbiosis and increased lipopolysaccharide (LPS) production. In turn, LPS causes skeletal muscle damage via the gut-liver-muscle axis. Consequently, LPS and obesity play a role in the induction of sarcopenia [183]. Fecal microbiota transplantation can be used in the future to treat NAFLD. It seems that fecal microbiota transplantation has a positive effect on gut microbiota disorders in these patients [184]. Furthermore, a combination of nutritional modification and increased physical activity contributes to a reduction in the intestinal dysbiosis characteristic of patients with NAFLD [185].

## 5. Conclusions

Many factors are shown to be common in the pathophysiology of NAFLD and sarcopenia. Given existing studies, it is impossible to say whether sarcopenia is a risk factor or a consequence of NAFLD. However, there is undoubtedly a link between the two due to the presence of common pathophysiological factors. More studies are needed to clearly define the cause-and-effect relationship between these entities so that it will be easier to select prevention and therapy for these patients. In addition, it is worth noting the introduction of physical activity that is tailored to the patient’s capabilities, as well as dietary modifications depending on the patient’s comorbidities and dietary preferences. Ultimately, there is a lack of data that clearly identifies lifestyle changes as adjunctive therapies for primary treatment in patients with NAFLD associated with sarcopenia.

## Figures and Tables

**Table 1:** Studies on the association between sarcopenia and NAFLD.

Study	Relationship between Sarcopenia and NAFLD	Limitations
Hong et al. (2013) [107]	A higher risk of NAFLD has been shown to occur in people with lower muscle mass.	Cross-sectional nature of the study—no causal relationship possible. No analysis of muscle strength and muscle fibre types. No information on confounding factors, e.g., the physical activity of patients and stimulants.
Petta el al. (2016) [94]	Liver fibrosis in NAFLD patients is 2-fold higher than in sarcopenic patients.	Cross-sectional nature of the study—impossibility to determine a cause-and-effect relationship. Lack of information on confounding factors, e.g., the physical activity of patients. Determination of sarcopenia based on skeletal muscle mass index measured by bioelectrical impedance analysis.
Lee et al. (2016) [95]	Liver fibrosis in NAFLD patients is 2-fold higher than in sarcopenic patients.	Cross-sectional nature of the study—no causal relationship possible.
Cabrera et al. (2016) [108]	In mice with induced NAFLD, an association of reduced IGF-1 with reductions in muscle mass and muscle strength is found. There appears to be a link between reduced IGF-1 and the pathogenesis of NAFLD-associated sarcopenia.	Mouse study.
Koo et al. (2017) [96]	The incidence of significant fibrosis was higher in patients with sarcopenia than in those without sarcopenia. Low muscle mass is associated with the histological severity of non-alcoholic steatohepatitis.	Sarcopenia was defined as a ASM/body weight (ASM%)
Saeki et al. (2020) [102]	Osteosarcopenia and frailty were closely associated with impaired physical performance in patients with CLD.	No analysis of confounding factors such as nutrition or physical activity. No assessment of the correlation between osteosarcopenia and frailty syndrome. No analysis of pharmacotherapy on the development of osteosarcopenia and frailty syndrome.
Tantai et al. (2021) [97]	Sarcopenia was independently associated with a 2-fold increased risk of death in patients with cirrhosis.	
Han et al. (2022) [109]	TNF- α is associated with cirrhosis-associated sarcopenia.	Retrospective analysis. A detailed analysis of the mechanism of TNF-α-induced sarcopenia in cirrhosis is lacking. The sample size was small.

**Table 2:** Myokines.

Myokine	Function
Miostatin	muscle hyperammonemia results in increased expression of myostatin and reduced activation of Nuclear Factor kappa B (Nf-B). As a result, impaired muscle protein synthesis occurs [159] is a negative regulator of muscle mass, thus playing a key role in the development and maintenance of skeletal muscle mass [160] myostatin appears to be one of the mediators of insulin resistance-induced skeletal muscle atrophy [162] high myostatin levels in patients with cirrhosis are correlated with lower survival rates [163]
Irisin	is produced in response to physical exertion [165] is responsible for fat browning and thermogenesis [165] has anti-inflammatory effects in the liver and activates hepatic autophagy [166] it is possible that irisin treatment may reduce age-related skeletal muscle fibrosis [169] irisin increase through physical activity may reduce inflammation in NAFLD [170]
Adiponectin	is one of the most common adipokines in human plasma. [171] is mainly produced and secreted by white adipose tissue [171] induces fatty acid oxidation and glucose utilisation [172] plays a hepatoprotective role in liver inflammation [173] it appears that alterations in its levels may contribute to the development of the metabolic syndrome and the development and progression of NAFL to NASH, as well as NASH-induced cirrhosis [174,175]

## Data Availability

No new data were created or analyzed in this study. Data sharing is not applicable to this article.
